# Thoughts on Medical Progress

**Published:** 1874-03

**Authors:** T. D. Washburn

**Affiliations:** Hillsboro, Ill.


ART. II.—*Thoughts on Medical Progress.* By T. D. WASHBURN, M. D., Hillsboro, Ill.

Medical Science more than any other, deals with the doubtful, the intricate and insoluble. The true man, who has most faithfully pursued the study of medicine—has diligently mastered the broad basis of facts, upon which the more elegant superstructure is founded—the man of broadest views, of deepest research, of wide practice and observation you will ever find modest, reticent and open to correction and instruction.

He may have well established convictions, certain landmarks well defined and immovable, but circumstances, contingencies and complications are ever liable to develop and to modify his views or warp his judgment. Anatomy is the same now, as a century ago and once acquired, remains; its boundaries are fixed and lie open to every careful observer; but when *hypothesis* enters into the equation or problem as it often does in physiology, pathology and therapeutics, we hesitate to endorse even the truth, until confirmed by more observation and stronger testimony. In Pathology one finds *such* a lesion, another in a like case finds another, and not until a vast variety of observations have been recorded and all the modifying causes presented, are we prepared to decide.

The wonderfully *diverse* views, which have been advocated in therapeutics, only substantiate the still more diverse views of pathology. Even the *function* of various organs—take for instance the kidney—hardly two will agree as to its duties—its legitimate and vicarious work; the same holds as to the liver, though much of their *particular* work has been demonstrated; but when the pancreas and spleen are interrogated, we are almost on unexplored ground—their positive functions remain to be found out.

Even the nervous system, that we supposed pretty well defined so far as its laws and mode of action were concerned, seems likely to be unceremoniously changed by the great investigator and acknowledged leader in this department, Brown-Sequard. Look again at the medical changes in practice (which of course presumes a change in theory) that have taken place in a score of years:—the lancet—ignored—antimony placed under ban—cold water brought to the front—diet, air and exercise superseding the pills, powders and mixtures, against which the *natural* man has so often rebeled. The contents of even a *regular* pair of saddle bags—how strangely different; *veratria, gelsemium* and *aconite* for fever, in place of *ipecac, nitre,* and *antimony.* Podophylin, leptandria and colycinth in place of rheubarb, calomel and jalap for cathartics. Bromide, sulphite and chlorate of potassa in place of the nitrate, sulphate and acetate, and for nervines, anticeptics, &c. Chloral, carbolic acid, permangnate of potassa, glycerine and a half dozen others which never saw a medical trunk or pocket case a half score of years ago.

Where shall we bring up? Is Science fallible? But you find a corresponding progress in surgery and all collateral branches. Chemistry, Physiology and Materia Medica are constantly solving new problems and sometimes bringing strange phenomena and facts square up against our old notions and prejudices. Look to where the *cell* theory is leading us, which with the aid of the microscope is largely demonstrable.

One little disturbing element, like this, brought to light, and we cannot tell where its influence will end.

The discovery of the circulation revolutionized the whole theory and practice of medicine. Liebig's theory of animal heat changed our therapeutics vastly. The immortal Jenner gave us a principle that the world does not appreciate and medicine has not yet seen half its benificient results. Seldom is a single grand discovery announced without many minor ones following. The finding of a new planet in proximity to another must change all previous astronomical calculations and results. A new force in any science that has remained a long time hidden or obscure requires a new adjustment of old forces, a reconstruction in all the minutiæ; so too in medicine, every new fact brought to the surface requires a revision both of theory and practice, and medical science more than most others is liable to these disturbing forces, for its intimate connection with mind, which reaches towards the infinite, forever forbids its becoming a *complete* science.

Mere matter, its composition and laws, can be pretty well defined and demonstrated, but complicate matter with mind, and study them as they must be studied as complex organisms and forces; and while we establish a large pile of facts and elucidate many of its laws, still there is always a *vast* field *away beyond*, which eludes our most vigorous grasp, and the most erudite and gifted fail to comprehend.

Thus we find an *unknown* in medicine, which ever stimulates the noblest and wisest; now and then surrendering a little fragment to the more patient and indefatigable, but the *limits* can never be reached.

This need not discourage the true student, or the ambitious; enough has been learned to found a noble system and establish a true science which has become the solace of the unfortunate, and a glorious boon to humanity, but *finite* beings must be content with *finite* science.

Infinity will ever be so conspicuously labeled on all that pertains to mind, that our highest nature and noblest efforts cannot fail to realize that our vision is circumscribed, and that "secret things are reserved for the Most High." These fine spun theories on "organic dynamics" attempting to solve all nerve force and life force on a more *material* basis, this exquisite and transcendent mechanism and combination of mind and matter, by the great laws of physics; bringing down the loftiest and noblest efforts of the great first cause to the mere demonstration of the *finite*, offends our sense of manhood, and grates harshly on our earliest, most deep-rooted and best proven convictions.

I have alluded to some of the *changes*, which have taken place in a single generation in connexion with the profession of medicine, but these are no greater than we witness in other matters. In our recollection the Railroad and Steamboat has become a fact, while the Telegraph is a thing of yesterday; the improvements in *printing, mining, dyeing, photography* and various other arts are patent to all. Under these circumstances two dangers are imminent, yes, always present, I mean the advocates or disciples of the *two* extremes; one ever yearning for the most advanced and *possible*; the other content to go *slow*, with the well proven, long established and *venerable;* always in the rear, they attempt to protect the grand army from any flank movement, or incursion from the new lights and undisciplined empirics, who are ever ready to seize any advantage and project an attack where they discover a weak point. As to facts, we are forced to acknowledge very much is submitted for our acceptance and adoption, both in Class Books, Journals and by living Teachers, which is far from being established.

The manifestation of new phenomena necessarily involves new hypotheses and men are so variously constituted, that a plus or minus imagination makes a wide difference in regard to intellectual results, so that the student as well as the advanced practitioner has to be constantly on his guard in accepting theories as well as deductions.

Right here, allow me to drop the remarks that if the student of medicine is well fortified with a thorough English education, natural philosophy, botany, chemistry and higher mathematics with a partial knowledge of Latin and Greek; if he selects the best medical school and attends three courses of lectures and three full years of study, he is not likely to mount every hobby and pet theory of every medical pretender and empirical vagabond. It is the emphasis we place on thorough education that has ever distinguished the *regular* from the *irregular*, and still constitutes the marked feature between science and empiricism.

Fortunately up the present day, the tendency is mostly in the right direction. The people are finding out, that they cannot afford to be humbugged; it costs too much, and every science and profession in their advanced reconstruction, are boldly thrusting the rotten and untenable out, holding fast only to the real, the most probable and best proven. Our analytical resources have become so great, the statistics so numerous, the conviction so deep in favor of the best, in all that pertains to education and science, that clap-trap, gas and pretension are forced to take back seats. I need not cite you to Harvard, Yale and our own Chicago, in testimony of this assumption; the statistics of the old country, everywhere, as well as our own favored land declare the fact, that ignorance, vice and mortality go hand in hand and are as inseperably connected as cause and effect.

Two things, we as a people are excessively fond of, our lives and our purses, and when the masses become impressed with the close connexion between intelligence and longevity, health and prosperity, the shams and tricksters who have cheated them out of both must go to the wall.

Our seminaries, colleges and all educational and scientific institutions in the land are only reflecting the wonderfully advanced views on popular education, which have taken hold of the whole country, nor will medicine constitute an exception.

For the last twenty years we have heard discussed among the profession, the question of Medical Education and the necessity of raising the standard.

Men of highest eminence attempted to establish a standard. The National Association, and finally a convention of Teachers sought to solve the problem; year by year they gradually approximated, but never completed their model. The latitude or longitude would cause a variation—the iso-thermal line could not be drawn. The competitive influence of locality, facilities, expenses and imposing names, all had their weight. In the meantime individual members rose to prominence by their untiring labors in different departments and increased the agitation and demand.

The war gave special emphasis to surgery, and the immense storehouse of facts, both in surgery and practical medicine have been so admirably collated—the hygienic and sanitary conditions so faithfully observed and the deficiencies so promptly met, that brother Jonathan at once presented himself as the peer of all his teachers and took front rank in everything pertaining to military, surgery, field and hospital practice.

Our rapid growth creating the demand, our increased wealth furnishing the means, the broadest liberty of thought and action; the stimuli of numerous Journals and multiplied schools, have gradually forced men to the front, and men are only the principles, or the combined forces they represent, either in medicine, politics, or religion. All this time chemistry, physiology and therapeutics have been pushing their investigations and accumulating facts. Social and sanitary questions have been broached calling upon us, to give in our evidence. Science and scientists have loomed up in vast proportions, flashing new light on old dogmas, and shaking the very foundations of moss-covered opinions, and much, that a score of years ago, was but a feeble cry, or undeveloped want, has become now a demand, a necessity of society and the times. So when Chicago leads off with her new classification of studies, and increased advantages, adopting what one of her more prominent sons had long sought, nothing more natural than dignified Harvard with her abundant means, should project the standard a little higher and follow in her wake; nor will Philadelphia long hesitate; already her stalwart Gross has rung out the demand of the times and the response of his listeners showed their hearty unison; a few distinguished centres thus taking the advance, and the less favored will soon follow.

Of all this we are proud; but *success* is not always stimulating, too often anæsthetic. We are far from having reached such a height that we can afford to relax our efforts, and look complacently on laggards or opponents; there are other victories to be won. An advanced position only reveals *new* difficulties and obstructions, or gives us a better view of *old* ones. A mountain scaled and summit reached brings to view not only new landscapes, but often other summits still less accesible.

There are some very practical questions far from being settled, dividing the profession which should be met in the spirit of kindness and frank discussion.

Our Code of Ethics (which I presume was not made for all time) must be modified or amended.

Our relations to the Public and the Public Press must be defined. Whether the *suppression* of quackery depends on higher attainments and nobler devotion to the regular profession and its practice, or whether the people themselves should be taught more thoroughly the nature of disease, the elements of health and laws of life to help them escape the clutches of Quackery, are among the mooted questions; certainly there is magnanimity and intelligence enough to settle amicably all these matters. In the spirit of true scientists let us marshall the facts, and bow to their logic.

